# External auditory canal haemorrhage as the first sign of internal carotid artery pseudoaneurysm, a rare case: a case report

**DOI:** 10.11604/pamj.2020.37.163.21968

**Published:** 2020-10-15

**Authors:** Laina Ndapewa Angula, Le Sun, Ning Fang, Xin Wang

**Affiliations:** 1 Peking Union Medical College Hospital, Beijing, China

**Keywords:** Internal carotid artery, pseudoaneurysm, haemorrhage, epistaxis

## Abstract

Assessing the cause, severity of bleeding and strategies to control bleeding is crucial. We describe a rare case of a patient who was presented with epistaxis and left ear haemorrhage, as a probable complication of a ruptured internal carotid artery pseudoaneurysm. The massive haemorrhage compelled blood transfusion and clinical intervention. The diagnosis of internal carotid artery (ICA) pseudoaneurysm measuring 2.9 cm x 3.7 cm was concluded by computed tomography. Several coils were used to embolize the internal carotid artery pseudoaneurysm and arrest the bleeding with the guidance of an angiography. Coiling the pseudoaneurysm is highly recommended. Yet, the best methods to completely treat aneurysm are still in question. After the clinical intervention, the patient remained symptom-free and no episodes of bleeding were noted.

06 Mar 2021: Corrigendum: External auditory canal haemorrhage as the first sign of internal carotid artery pseudoaneurysm, a rare case: a case report. Pan Afr Med J. 2021;38:239. doi: 10.11604/pamj.2021.38.239.28017

## Introduction

Vascular lesions are severe complications caused by an invasive tumour, blood dyscrasia, penetrating trauma or blunt, or iatrogenic origin [1]. Pseudoaneurysm is an unusual vascular complication as a result of a partial injury of an arterial vessel wall, which causes blood flow via the laceration into the neighbouring tissues. This continuous leakage results in a slowly enlarging mass that results in a pseudoaneurysm over time [2]. Various clinical manifestations of bleeding from fatal to moderate can take place resulting in cranial or central nerve deficit. Considering its life-threatening course, the attending doctor has a limited time to identify and treat these lesions. We present a case of epistaxis and massive haemorrhage of the ear due to internal carotid artery pseudoaneurysm, a complication likely stemming from surgical debridement of necrotic fasciitis of the left side of the neck performed previously.

## Patient and observation

**Figure 1:**
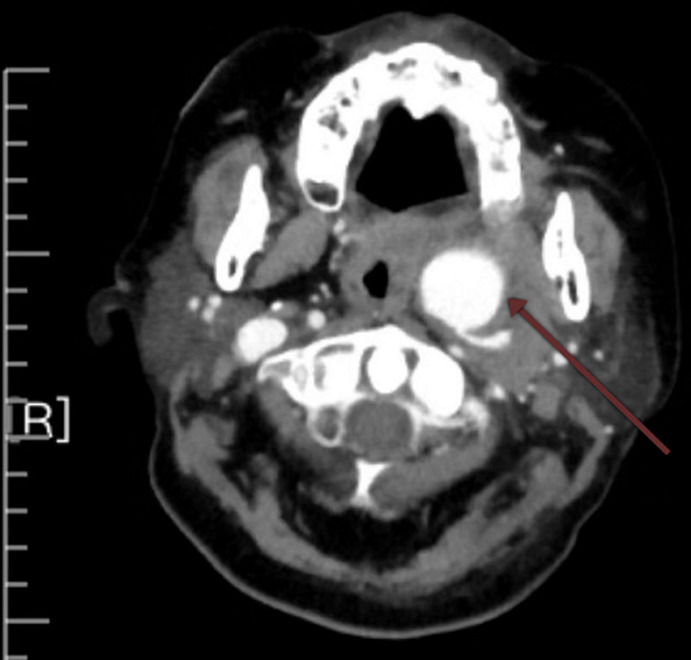
neck CT showing a high-density shadow measuring 2.9 × 3.7 cm at the area of the ICA, parapharyngeal space and pharyngeal space

A 66-year-old woman presented with epistaxis and haemorrhage of the left ear without any obvious inducement for a period of one week. The patient has a 7 year history of hearing loss, mastoiditis, left tympanic membrane perforation and cervical necrotizing fasciitis which prompted a surgical debridement. A pseudoaneurysm was not observed during that time. The coagulation profile revealed that the haemoglobin level on admission was 60 g/L. An otoscopic examination revealed a blood-stained left external auditory canal. The tympanic membrane was intact. A laryngoscope examination revealed blood stained secretions in the posterior pharyngeal wall. Left pharyngeal oedema and a swollen uvula was observed. Neck contrast-enhanced computed tomography (CT) scan showed a slightly high-density shadow visible in the left parapharyngeal space, pharyngeal space and internal carotid artery area measuring 2.9 cm x 3.7 cm (Figure 1). Oropharynx and laryngopharynx were compressed and narrowed.

**Figure 2:**
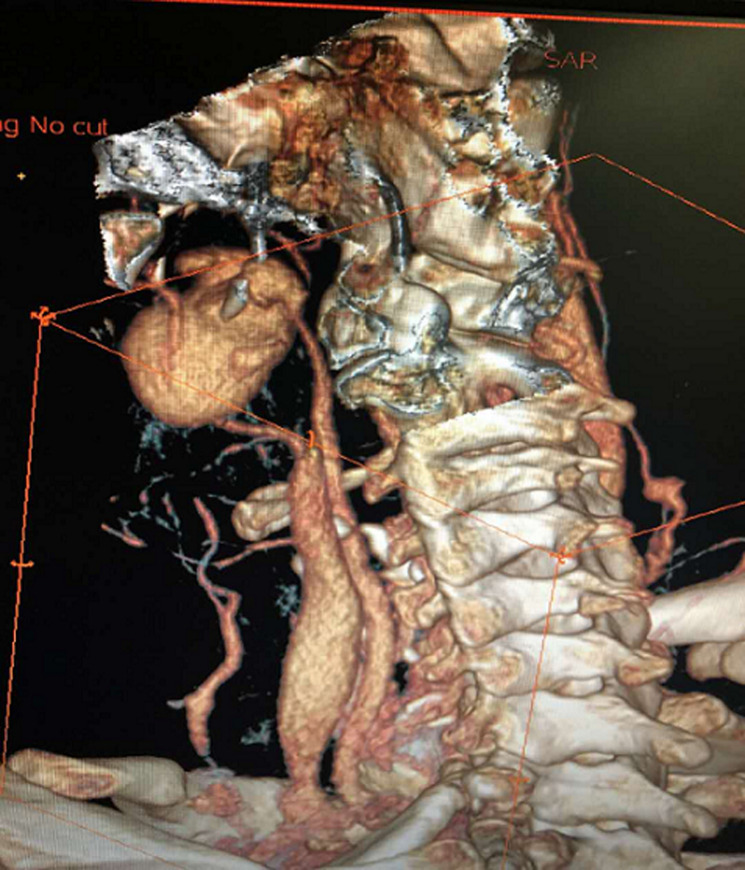
preoperative angiography of the left ICA showing the pseudoaneurysm

**Figure 3:**
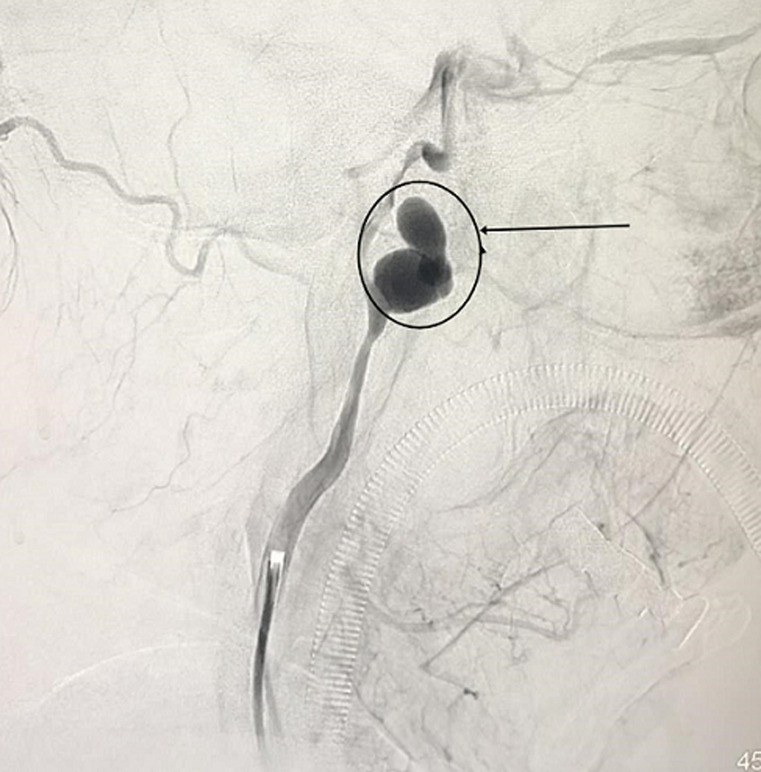
preoperative angiography of the left ICA showing the pseudoaneurysm projecting laterally

**Figure 4:**
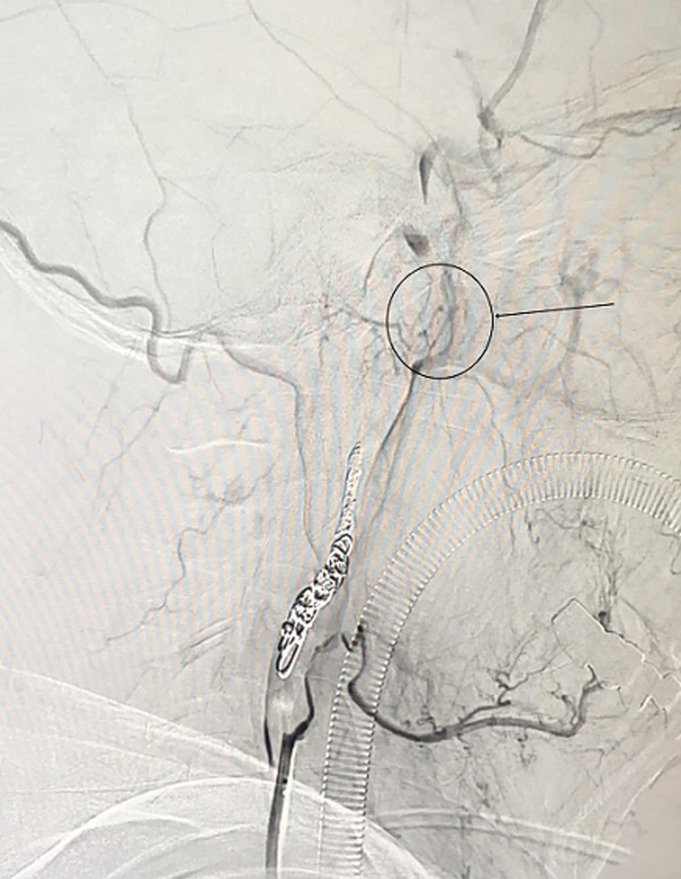
postembolization angiography revealed a complete exclusion of the pseudoaneurysm

The patient underwent ICA coil occlusion. The angiogram was obtained to confirm the pseudoaneurysm and check the flow (Figure 2 and Figure 3). With the help of a 5F catheter and a guide wire, the pseudoaneurysm was successfully embolized using coils. Postembolization angiography revealed a complete exclusion of the pseudoaneurysm (Figure 4). The patient was given a blood transfusion. Follow-up evaluations revealed no recurrent episodes.

## Discussion

Pseudoaneurysm treatment using coil embolization from the external auditory canal is effective and a secure option to usual surgical ligation of the affected artery, as illustrated in our case. No patient has been reported with associated functional impairment after coil embolization procedure. In our case, iatrogenic and injury are the possible causes of the pseudoaneurysm. The diagnosis of pseudoaneurysm should highly focus on the past history of injury and also a physical examination. Physicians should refrain from using a diagnostic needle aspiration due to the risk of bleeding and the related dilemma of dealing with such bleeding in an orifice setting.

Contrast-enhanced computed tomography (CT) can illustrate a vascular enhancement with a round lesion. It can outline the site and degree of the lesion and also disclose any coexisting pathologies which cannot immediately be diagnosed clinically. Still, CT has inadequate detective sensitivity in some cases due to artefact from metallic fragments. Apart from the limitations of a CT scan, we still pursue to utilize it as a basic screening option [3,4]. The clinical diagnosis of a pseudoaneurysm was confirmed by the carotid angiogram before any kind of treatment is given. Angiography is more preferred when the lesion has been assessed and endovascular treatment is a reasonable alternative.

Endovascular, surgical and conservative management is the treatment options for the pseudoaneurysm [5]. We opted out the duplex-guided thrombin injection approach as its distribution of the thrombin is not well regulated and may result in some complications [6], despite the fact that it may also be a useful option to maintain a pseudoaneurysm [4]. Catheter-based embolization is a secure, rapid and effective method for the treatment of pseudoaneurysm. Various agents are used for embolization therapy, such as gelatin sponges and coils [5]. Benefits of an endovascular method include avoiding wound associated complications and facial scars. In our hospital, a pseudoaneurysm its branches are treated by occlusion of the involved artery via coils embolization over the neck of the pseudoaneurysm due to the fact that the artery is relatively tiny.

## Conclusion

An ICA pseudoaneurysm should be taken into consideration if there is extreme epistaxis and a past history of a neck injury, even though it occurred many years ago. Pseudoaneurysm from the external auditory canal and its branches are rare in the head and neck. This case illustrates that a pseudoaneurysm can be treated by coil embolization in a secure, fast and beneficial manner.
