# Corrigendum: Laina Ndapewa Angula *et al*. External auditory canal haemorrhage as the first sign of internal carotid artery pseudoaneurysm, a rare case: a case report doi: 10.11604/pamj.2020.37.163.21968

**DOI:** 10.11604/pamj.2021.38.239.28017

**Published:** 2021-03-06

**Authors:** Laina Ndapewa Angula, Le Sun, Ning Fang, Xin Wang

**Affiliations:** 1 Department of Otolaryngology, First Hospital of Jilin University, Changchun, China,; 2 Peking Union Medical College Hospital, Beijing, China

**Keywords:** Corrigendum, internal carotid artery, epistaxis, haemorrhage, pseudoaneurysm

## Abstract

This corrigendum corrects article “External auditory canal haemorrhage as the first sign of internal carotid artery pseudoaneurysm: a rare case” and its publication reference i.e. The Pan African Medical Journal. 2020; 37:163. Access corrected manuscript Pan African Medical Journal. 2020; 37: 163. doi: 10.11604/pamj.2020.37.163.21968. PubMed PMID: 33425196. PubMed Central PMCID: PMC7757232. Epub 2021/01/12. eng.

## Corrigendum

The original version of this article [1] had authors' affiliations reversed in the article citation on PubMed. The affiliations of the following authors including the corresponding author: Laina Ndapewa Angula, Ning Fang and Xin Wang have been corrected by inverting author's affiliations as follows; “Peking Union Medical College Hospital, Beijing, China” to “Department of Otolaryngology, First Hospital of Jilin University, Changchun, China”.
